# Using deeply time-series semantics to assess depressive symptoms based on clinical interview speech

**DOI:** 10.3389/fpsyt.2023.1104190

**Published:** 2023-02-14

**Authors:** Nanxi Li, Lei Feng, Jiaxue Hu, Lei Jiang, Jing Wang, Jiali Han, Lu Gan, Zhiyang He, Gang Wang

**Affiliations:** ^1^Beijing Key Laboratory of Mental Disorders, National Clinical Research Center for Mental Disorders and National Center for Mental Disorders, Beijing Anding Hospital, Capital Medical University, Beijing, China; ^2^Advanced Innovation Center for Human Brain Protection, Capital Medical University, Beijing, China; ^3^Anhui iFLYTEK Health Co., Ltd., Hefei, China

**Keywords:** depression, mood disorder, psychiatric assessment, semantic, time-series, natural language processing

## Abstract

**Introduction:**

Depression is an affective disorder that contributes to a significant global burden of disease. Measurement-Based Care (MBC) is advocated during the full course management, with symptom assessment being an important component. Rating scales are widely used as convenient and powerful assessment tool, but they are influenced by the subjectivity and consistency of the raters. The assessment of depressive symptoms is usually conducted with a clear purpose and restricted content, such as clinical interviews based on the Hamilton Depression Rating Scale (HAMD), so that the results are easy to obtain and quantify. Artificial Intelligence (AI) techniques are used due to their objective, stable and consistent performance, and are suitable for assessing depressive symptoms. Therefore, this study applied Deep Learning (DL)-based Natural Language Processing (NLP) techniques to assess depressive symptoms during clinical interviews; thus, we proposed an algorithm model, explored the feasibility of the techniques, and evaluated their performance.

**Methods:**

The study included 329 patients with Major Depressive Episode. Clinical interviews based on the HAMD-17 were conducted by trained psychiatrists, whose speech was simultaneously recorded. A total of 387 audio recordings were included in the final analysis. A deeply time-series semantics model for the assessment of depressive symptoms based on multi-granularity and multi-task joint training (MGMT) is proposed.

**Results:**

The performance of MGMT is acceptable for assessing depressive symptoms with an F1 score (a metric of model performance, the harmonic mean of precision and recall) of 0.719 in classifying the four-level severity of depression and an F1 score of 0.890 in identifying the presence of depressive symptoms.

**Disscussion:**

This study demonstrates the feasibility of the DL and the NLP techniques applied to the clinical interview and the assessment of depressive symptoms. However, there are limitations to this study, including the lack of adequate samples, and the fact that using speech content alone to assess depressive symptoms loses the information gained through observation. A multi-dimensional model combing semantics with speech voice, facial expression, and other valuable information, as well as taking into account personalized information, is a possible direction in the future.

## Introduction

Depression is a common mental disorder characterized by a persistently depressed mood, a loss of pleasure or interest in activities, and other associated symptoms. The World Health Organization (WHO) reports that approximately 5% of adults worldwide suffer from depression. Depression is a major contributor to the global burden of disease (1). Appropriate assessment plays a key role in clinical practice with Measurement-Based Care (MBC) being recommended for depression management in several clinical practice guidelines (2). Symptoms assessment is one of the most important dimensions with a number of scales available in depression evaluation.

The Hamilton Depression Rating Scale (HAMD) is the most commonly used assessment tool to probe the presence of depressive and associated symptoms, and is considered the “gold standard” of depression measurement (3), and has been used to establish the criteria for the severity level of depressive symptoms (4). Numerous versions of the HAMD exist, and a 17-item version of the Hamilton Depression Rating Scale (HAMD-17) is the most classic and widely used version (5). Each item of the HAMD-17 examines a subsymptom of depression, and some of the items are formed into a factor structure. The psychopathology and symptom clusters can be specifically characterized by factor analysis. The HAMD is not only used for depression, but can also be used for a variety of diseases such as bipolar disorder (BD), neurological disorders and other medical conditions with depressive symptoms. Therefore, a clinical interview incorporating HAMD-17 is appropriate for broad questioning and assessing depressive symptoms.

As the HAMD-17 is a hetero-rated scale, it requires a trained rater, with specific expertise, sufficient knowledge of the scale, and reliable accuracy. The scale was originally designed to be completed after an unstructured clinical interview. Although semi-structured interview guides are available (6) that record only the score and not the interview process, there is a risk of bias in obtaining accurate scores based on the unreviewable interview. In addition, due to medical resource constraints, there is a need for more professionals to conduct regularly high quality assessments in a real-world clinical setting.

Natural language processing (NLP) is a branch of AI that focuses specifically on understanding, interpreting, and manipulating large amounts of human language and speech data. Since the 1980s, NLP has combined computational linguistics with statistical machine learning and Deep Learning models in order to take unstructured, free-form data and produce structured, quantitative linguistic outputs. With the growth of available public data, NLP technology based on time-series learning has grown significantly in recent years (7), particularly in medicine, where more and more research is demonstrating the value of Deep Learning-based NLP (8). The use of Deep Learning-based NLP in medicine is particularly useful in prediction and reverse distillation based on regular medical records for risk assessment (9), thus, using time-series semantic information by simulating clinical decision-making for risk forecasting (10). Resent research has shown that NLP has the ability to perform highly repetitive manual tasks consistently and to integrate and compute knowledge efficiently compared to human beings, opening up more opportunities for the use of NLP in practical applications. The assessment of specific depressive symptoms is a suitable application for NLP. The process of assessing depressive symptoms, because of its clear purpose and specific content, depends on specific expertise and information integration based on the interview. In addition, HAMD-17 has provided a framework for interviewing and evaluating depressive symptoms, as well as normative classification criteria for the severity level of depressive symptoms, which meets the need for the application of Deep Learning techniques.

Numerous studies have focused on and attempted to apply NLP technology approaches to detect and evaluate depression; however, they have mostly extracted data in the form of written text (11), which differs significantly from oral text. The data used for NLP has been extracted from electronic health records (12) and social media (13). The text is either processed by a doctor or without any professional processing, and the semantic density of the accessible information is sparse compared to specific interviews about depressive symptoms. Therefore, it is valuable to apply NLP techniques directly to the interview text for the assessment of depressive symptoms in order to build a framework of depressive semantic model, thus providing the opportunity to bring AI technology into psychiatric clinical practice in the future.

The aims of this study include: (1) to construct a task-oriented algorithmic model using text from specific clinical interviews, and to validate the feasibility of Deep Learning-based NLP for depressive symptoms assessment. It should be noted that the model construction and the core algorithm are not the entire purpose of this study, but rather a methodological approach; therefore, its introduction is presented in the Data Analytic Strategy section, (2) to validate the proposed time-series semantic algorithm model, and to measure the performance of classifying the depressive symptoms severity level.

## Materials and methods

Data for this study were derived from two clinical research projects, one is about emotional recognition among patients with depression, and the other is regarding identifying unipolar and bipolar depression using speech. The Ethical Committee of Beijing Anding Hospital has approved both projects. Each participant was asked to sign a written informed consent before data collection.

### Participants

In this study, 329 participants with Major Depressive Episode (MDE) were recruited at the Beijing Anding Hospital inpatient or outpatient departments from September 2020 to July 2022. At the time of enrolment, 233 participants were diagnosed with Major Depressive Disorder (MDD), diagnosed by experienced psychiatrists according to the ICD-10 for inpatients and the Mini International Neuropsychiatric Interview (MINI, version 7.0.2) for outpatients. In addition,, 96 participants were diagnosed with bipolar disorder having concurrent depressive episodes (BDD) using the MINI. All participants met the inclusion criteria, which included: (a) age between 18 and 65 years, (b) speaking Chinese without obvious dialect, (c) educational level of primary school or above, (d) understanding and cooperating with the research content. Exclusion criteria included: (a) a diagnosis of schizophrenia, schizoaffective disorder or other mental disorders, (b) a history of organic brain disease. All 329 participants had a mean age of 34.1 (SD: 12.4) years, ranging from 18 to 64 years, and 66.0% (*n* = 217) of the participants were female. The participants’ current MDE lasted 28.8 (SD: 47.9) weeks, with a mean HAMD-17 score of 20.2 (SD:5.72).

### Procedure and measures

Each participant was asked to complete a face-to-face clinical interview using the HAMD-17 with an audio recording. All interviewers were trained and scorer reliability was maintained to ensure the quality of the interview. A number of standard phrases were developed in the interview outline which were used to locate the interview content and facilitate text processing. All interviews were conducted in special test rooms with no noticeable background noise. The recording device was either an audio recorder (brand and model: iFLYTEK SR502) or a smartphone (brand and model: honor 9X). The recording device was placed approximately 50 cm away from the participant.

Basic demographic information and a brief medical history were collected before the audio recording. The HAMD-17 was used in this study. In this version, each item is scored from 0 to 2 or from 0 to 4, and the total score ranges from 0 to 52. We defined the cut-off points, and the severity levels of the depressive symptoms as follows: >24 = severe depression, 18–24 = moderate depression, 8–17 = mild depression, <8 = euthymia. A total of 387 audio recordings were collected during the study, as 58 of the 233 participants with MDD received the same secondary clinical interview 4 weeks after the initial interview. Finally, according to the HAMD-17 total score, 46 audio recordings were classified as euthymia, 102 were classified as mild depression, 160 were classified as moderate depression, and 79 were classified as severe depression.

### Data preprocessing

The initial form of data collected was an audio recording of the clinical interview between the doctor and the patient. Considering the content composition of the audio, the data was pre-processed in four steps: speaker diarization, role identification, speech recognition, and item-centered classification. The final data output is presented as a structured Chinese text of the doctor-patient dialogue (see Supplementary Appendix 1 for more details). The speaker diarization and speech recognition technology are supported by the iFLYTEK open platform.^1^ Role identification was used to distinguish between doctor and patient through a rule-based approach. Two main rules are used: (1) after building a library of doctor question sentences, the edit distance is calculated from the input data to determine whether the role is a doctor, (2) a keyword database of question sentences was summarized and constructed, the identities of the doctor and the patient are determined by calculating the frequency of keywords throughout the conversation. An item-centered classification scheme, based on the temporal analysis of bidirectional long and short-term memory (BiLSTM) (14), was used to cut the text and extract the content related to the corresponding HAMD-17 item using a pair of question and answer (Q&A) sentences as the input of each temporal step as well as the corresponding item names as the output. The topics measured by each item of HAMD-17 are defined and described in the model as *scene*.

### Dataset settings

The dataset consisting of 387 audio recordings was randomly grouped into training set and test set in a ratio of 7:3, with no significant differences in the overall distribution of the HAMD-17 total score. Thus, 114 audio recordings were put into the test set, and the corresponding severity level of depressive symptoms was distributed as 23 euthymia, 50 mild depression, 30 moderate depression, and 11 severe depression. Additionally, 273 audio recordings were put into the training set, and the distribution of the corresponding severity level of depressive symptoms is 23 euthymia, 52 mild depression, 130 moderate depression, and 68 severe depression. The detailed distribution is shown in Table 1.

**TABLE 1:** Distribution of the number of audio data in training set and test set.

Depression	Number of audio		
severity level	Sum of total (387)	Training set (273)	Test set (114)
Euthymia	46	23	23
Mild depression	102	52	50
Moderate depression	160	130	30
Severe depression	79	68	11

### Model algorithms

Applying the level-classification measurement of depressive symptoms, a time-series semantics model based on multi-granularity and multi-task joint training (MGMT) was customized in this study. A brief introduction of MGMT is provided as the model and is being proposed for the first time.

#### General framework

The clinical interview speech is in the form of a doctor-patient dialogue which has a certain temporal development pattern. A quantitative scheme based on the time-series is used in this algorithm. For any given audio dialogue, after data pre-processing, the entire text of one scene is represented by X, $\text{X}=\left\{{\text{x}}_{\text{i}}^{\text{j}}:0<\text{j}<L,0<\text{i}=17\right\}$, in which *i* is the subscript of the scene, *j* is the text subscript of each scene, and L refers to the maximum text length. The text of each scene is encoded using Bidirectional Encoder Representation from Transformers (BERT) (15), [CLS] output, a token with no obvious semantic information, is taken as the text embedding of each scene, H is referred to as scene coding, and H={*h*_*i*_,0 < = *i* < = 17,*h*_*i*_ ∈ *R^d^*}, the *d* is 256 dimensions in this scheme. The scene granularity score is represented as S, where S={*s*_*i*_,0 < *i* < = 17,*s*_*i*_ ∈ *R*^1^}. The formulas are shown as follows:


H=Pool⁢(BERT⁢(X))

S=Softmax⁢(H)

While the H and S are calculated in the set of independent scene, BiLSTM is introduced to obtain information from the holistic dialogue and to concatenate scenes according to the time-series. The equations are present below. H^F^ ∈ *R^d^* and H^b^ ∈ *R^d^* are output vectors of the forward and backward LSTMs, respectively.


$${\text{H}}^{\text{F}}=\vec{L\,S\,T\,M}\,\left(H\right)$$

$${\text{H}}^{\text{b}}=\overset{\leftarrow }{L\,S\,T\,M}\,\left(H\right)$$

$$H={\text{[H}}^{\text{F}},{\text{H}}^{\text{b}}]$$

After performing Self-Attention, Multilayer Perceptron (MLP), and Softmax, combined with the obtained information from entire dialogue the depressive severity level and its probability from a holistic perspective are presented as P^e^, and ${\text{P}}^{\text{e}}=\left\{{\text{p}}_{\text{i}}^{\text{e}},0<=\text{i}<4\right\}$.

The final level is produced by the Decision Level Fusion competition. Equations are expressed as follows:


S⁢_⁢e2e⁢_⁢level=Softmax⁢(MLP⁢(Self-Attention⁢(H)))

S⁢_⁢scene⁢_⁢level=HAMD⁢score⁢(S)

Level=Decision⁢Level⁢Fusion⁢(s⁢_⁢e2e⁢_⁢level,S⁢_⁢scene⁢_⁢level)

**FIGURE 1:**
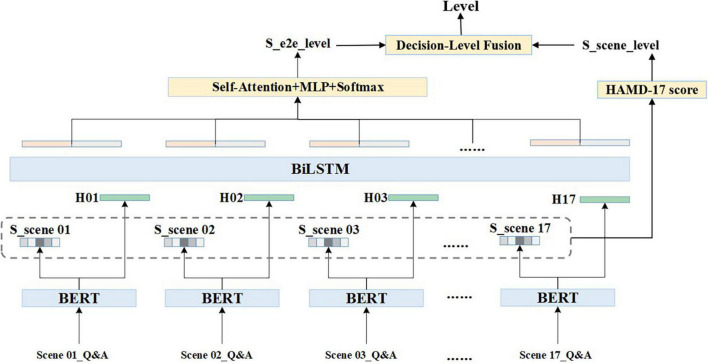
General framework of multi-granularity and multi-task joint training (MGMT).

Three strategies were used in the Decision Level Fusion module: (a) taking the S_e2e_level as the final level, (b) taking the S_scene_level as the final level, and (c) weighting the scene levels to the corresponding total score, reordering the levels based on corresponding probabilities, and taking the level with the highest probability as the final level. The general framework is illustrated in Figure 1.

#### Multi-task joint optimization

The optimization of MGMT is conducted using multi-task joint training with scene and HAMD scores. Due to dialogue content varying in complexity and the scene scores are unevenly distributed. The Focal Loss (16) and the GHM Loss (17) were used as the joint loss function for optimization. The idea of the Focal Loss is to reduce the weight of easily distinguishable samples (i.e., samples with high confidence) and to increase the weight of hard to distinguish samples, forcing the model to pay more attention to these hard to distinguish samples. Furthermore, considering the existence of many indistinguishable, mislabeled, and confused samples due to the lack of information the stability and optimization direction of the model will be affected. The GHM Loss is introduced to balance attention to the indistinguishable samples. Therefore, the final loss is the weighted and balanced result of the Focal Loss and GHM Loss, which is represented as:


$$\text{loss}={\text{L}}_{\text{FL}}+\gamma *{\text{L}}_{\text{GHM}}$$

In the above formulae, γ is hyperparameter configured to balance the weight of L_GHM_, which is specified by the distribution of the data set. Finally, the loss function of MGMT is present as the following equation where α and β are hyperparameters configured to balance the weight of the scene and the weight of the End-to-End separately.


$$\text{Loss}=\alpha *l\,o\,s\,s_{s\,c\,e\,n\,e}+\beta *l\,o\,s\,s_{e\,2\,e}$$

Considering that in an actual clinical setting, there may be some scenes that are incomplete or insufficient to provide valid information; therefore, each scene is masked with a probability of 5%. The score of the masked scene is set to 0 and the entire data is used as a new sample for training. This operation is intended to simulate the real interview process and to enhance the generalization ability of the model.

#### Model training and performance evaluation

A BERT-small model trained each model with 6 transformer block layers, with each block having a hidden size of 256 and 12 multi-head attentions. The models were trained on a Tesla V100 32G GPU with a training epoch of 100 and a batch size of 16. An Adaptive Moment Estimation (Adam) optimizer with an initial learning rate of 0.001 and a warm-up learning rate decay strategy was used during model training. A single layer BiLSTM was used for temporal aggregation training with a hidden size of 256. The hyperparameters α,β,*and*γ were set to 0.8, 0.2, and 0.2, and the maximum length (L) of each scene was set to 512.

Model performance was measured using an F1 score defined as the following formulae. True Positive (TP): judged to be a positive sample when in fact it is a positive sample. False Positive (FP): judged to be a positive sample when in fact it is a negative sample. False Negative (FN): judged to be a negative sample when in fact it is a positive sample. The F1 score can be interpreted as a weighted average of precision and recall, with values taken at an interval range of 0–1.


$$\mathrm{F1}=\frac{2\,T\,P}{2\,T\,P+\text{FP}+\text{FN}}$$

## Results

### Formatted text description

The mean number of interactive rounds of the total data were 85.5 (SD: 24.5), the mean number of words spoken by each participant was 1,100 (SD: 599) counts, and the mean length of the audio was 681 (SD: 210) seconds. As we used Automatic Speech Recognition (ASR) technology, the quality of the formatted text is measured using word correctness (Corr) and word accuracy (Acc). Corr = H/N, where H represents the number of correctly recognized words and N is the total number of recognized words. Acc = (H-I)/N, H and N have the same meaning as before, I represents the number of inserted non-existent words. The formatted text used in this study is of good quality, with a Corr of 94.60% and an Acc of 93.10%.

### Classification result

Three types of classification were used to assess the performance of MGMT in evaluating depressive symptoms, including: (a) a four-level classification of depression severity (severe depression vs. moderate depression vs. mild depression vs. euthymia), (b) a binary classification of mild depressive and severe depressive symptoms (severe depression and moderate depression vs. mild depression and euthymia), and (c) a binary classification of identifying the presence of depressive symptoms (severe depression and moderate depression and mild depression vs. euthymia). In addition, 96 of the 387 audio recordings were collected from participants with BDD, who had a different diagnosis from those with MDD, so the original test set (ALL) was divided into a dataset including only MDD (with 92 audio recordings) and a dataset including only BDD (with 22 audio recordings) in order to verify the robust of MGMT.

Using the ALL training set, MGMT with ALL test set (ALL-ALL) has an F1 score of 0.719 in the classification of the four-level depression severity, 0.884 in the binary classification of mild depressive and severe depressive symptoms, and 0.890 in the binary classification of identifying the presence of depressive symptoms. For only MDD included in test set (ALL-MDD), MGMT has an F1 score of 0.706 in the four-level depression severity classification, 0.913 in the binary classification of mild and severe depression, and 0.837 in the binary classification of identifying the presence of depressive symptoms. There is no significant difference in the F1 score between ALL-ALL and ALL-MDD. Using the test set only including BDD (ALL-BDD), the best result is obtained with an F1 score of 0.772 in the classification of the four-level depression severity and an F1 score of 0.955 in both binary classification models. The results are shown in Table 2.

**TABLE 2:** Performance of MGMT in depressive symptoms evaluation (ALL as training set).

Classification type	F1 score		
	ALL-ALL	ALL-MDD	ALL-BDD
Four-level depression severity classification	0.719	0.706	0.772
Mild/severe depression binary classification	0.884	0.913	0.955
Euthymia/depression binary classification	0.89	0.837	0.955

The specific values for the accuracy of the four-level depression severity classification are shown in Table 3. In ALL-ALL, MGMT has an accuracy of 69.57% (16 of 23) in identifying euthymia, 76.00% (38 of 50) in identifying mild depression, 73.33% (22 of 30) in identifying moderate depression, and 54.55% (6 of 11) in identifying severe depression. In ALL-MDD, the performance of MGMT is similar to that of ALL-ALL, with an accuracy of 66.67% (14 of 21) in identifying euthymia, 73.17% (29 of 41) in identifying mild depression, 70.83% (17 of 24) in identifying moderate depression, and 66.67% (4 of 6) in identifying severe depression. In ALL-BDD, MGMT is correct in identifying all of euthymia, and has an accuracy of 88.89% (8 of 9) in identifying mild depression, 83.33% (5 of 6) in identifying moderate depression, while having an accuracy of 40.00% (2 of 5) in identifying severe depression.

**TABLE 3:** Performance of MGMT in four-level depression severity classification of three test sets.

Depression	Accuracy (correct/total number of audio)		
severity level	ALL-ALL	ALL-MDD	ALL-BDD
Euthymia	69.57%	66.67%	100.00% (2/2)
Mild depression	76.00%	73.17%	88.89% (8/9)
Moderate depression	73.33%	70.83%	83.33% (5/6)
Severe depression	54.55%	66.67%	40.00% (2/5)

To present the results more clearly, the confusion matrix of the four-level depression severity classification of ALL-ALL is shown in Table 4. Misclassification occurs more frequently in the proximity category.

**TABLE 4:** Confusion matrix of the four-level depression severity classification of ALL-ALL.

Confusion matrix		Predict			
		Euthymia	Mild depression	Moderate depression	Severe depression
True	Euthymia	16	7	0	0
	Mild depression	8	38	4	0
	Moderate depression	1	3	22	4
	Severe depression	0	1	4	6

### Sensitivity analysis

To further verify the reliability of the above results, we used only a total of 199 audio recordings of MDD patients as training set data (MDD-MDD), removing those of BDD patients from the training set. The performance of MGMT is shown in Table 5. MGMT has an F1 score of 0.685 in the four-level depression severity classification with MDD-MDD, which is slightly lower than that being produced by ALL training set (ALL-MDD). MGMT has an F1 score of 0.902 in the binary classification of mild depressive and severe depressive symptoms, and 0.826 in the binary classification of identifying the presence of depressive symptoms.

**TABLE 5:** Performance of MGMT in depressive symptoms evaluation (MDD-MDD).

Classification type	F1 score
Four-level depression severity classification	0.685
Mild/severe depression binary classification	0.902
Euthymia/depression binary classification	0.826

Table 6 shows the performance of MGMT with MDD-MDD in the four-level depression severity classification, MGMT owns an accuracy of 61.90% in identifying euthymia (13 of 21), 73.17% in identifying mild depression (30 of 41), and an accuracy of 66.67% in identifying both moderate depression (16 of 24) and severe depression (4 of 6). There is a slight decrease in accuracy compared to ALL-MDD.

**TABLE 6:** Performance of MGMT in four-level depression severity classification (MDD-MDD).

Depression severity level	Accuracy
Euthymia	61.90%
Mild depression	73.17%
Moderate depression	66.67%
Severe depression	66.67%

## Discussion

The present study developed a time-series semantics model primarily based on multi-granularity and multi-task joint training. MGMT obtained information about depressive symptoms in various dimensions, performed well on the task of classifying the severity level of depressive symptoms, and demonstrated the feasibility of Deep Learning combined with NLP applied to psychiatric assessment.

Early studies of depressive speech using computational analysis were generally based on psycholinguistics, with measures characterizing lexical diversity, syntactic complexity and speech content. Word counting, at the level of vocabulary granularity, was the most direct method of representing the speech characteristics. Relevant studies have identified differences in the frequency of first-person singular pronouns, negative mood words, and positive mood words between depressed patients and healthy controls (18). These differences were also found in patients with different severity levels of depressive symptoms (19). Sentence-level analysis provides insight into cognitive-linguistic conditions through the sentence structure. Sentence structure changes are less significant in patients with depression than in those with schizophrenia or Alzheimer’s disease (20). Changes in speech at the sentence-level in depressed individuals are more likely to be summarized by a reduced number of words in a sentence and a decrease in overall speech activity. While these changes have been shown to correlate with attention and psychomotor speed performance; however, they are less correlated with depressive severity (21).

According to the results in Tables 3, 6, the different performance of MGMT in distinguishing depression severity is in line with the corresponding sample size. The accuracy of the classification is relatively low with a small sample size. The small sample size is reflected not only in the audio number rated as severe depression but also in the frequency of occurrence of the extreme point of each item, especially when the variables are measured on a five-point. In addition, the confusion matrix in Table 4 shows that confusion occurs more frequently in the proximity category. The model has not learnt the key point of classification through adequate samples, it is prone to misjudgment when encountering unfamiliar or rare text. This just further confirms the importance of having a sufficient number of samples with clear distinctions for model training. Although the severity levels are conventionally and strictly divided by the HAMD-17 total score, samples on either side of the cut-off value have high similarity and low discrimination. A difference of 1 point in the total score may not make a significant difference in the evaluation of depressive symptoms, although they belong to different severity levels of depressive symptoms. The better option in this situation is to use the HAMD-17 total score directly or to redivide the depression severity sub-intervals. Moreover, the clear differentiation between samples is reflected in different scores for each item, different combinations of item scores, and slight differences in the total score. It is difficult to obtain sufficient data to build an equally distributed training data in a clinical setting, while it is critical for Deep Learning-based NLP technology.

In addition to the sample size mentioned above, there is another limitation of this study. Audio recordings contain information from both text and voice, and voice characteristics are also considered valuable in the assessment of depressive symptoms. Studies have found differences between the voices of depressed and healthy people (22), and acoustic features are correlate with the severity of depressive symptoms and their variability (23). Several acoustic features are thought to correlate with depression (24), Machine Learning and Deep Learning techniques have been widely used in the studies of voice analysis (25). For the audio recordings in this study, we also performed some voice analysis. we conducted a multi-feature decision fusion classification model, including X-vector, the extended Geneva Minimalistic Acoustic Parameter Set (eGeMAPs) (26), wav2vec 2.0, and conformer ASR decoder features. However, the accuracy of the model in classifying four-level severity of depression is only 43.86%. We also attempted to construct a semantic-voice fusion model, and the performance of this model did not improve over the text-only model conducted in this study. Therefore, we have mainly confirmed the value of semantics in depression severity classification without adding voice analysis.

Agitation and retardation are observational items based on the patient’s behavior during clinical interview. Using speech text alone is impractical for observational assessment and may lead to bias in the assessment of depressive severity using the HAMD rating scale. The external performance of agitation is excessive physical activity with significant fidgeting, tension and excitement, and psychomotor retardation can be detected by speech volume, response duration, and movement changes. Simulating actual human judgment, performance can be captured by other forms of behavioral indicators, such as speech as mentioned above, as well as, facial expressions and gestures. Datasets consisting of facial expressions and gestures are available for clinical analysis, and several features are associated with depressive symptoms, which can be used to construct depression detection models (27). Multidimensional information helps to optimize the assessment of depressive symptoms and compensates for observational information (28).

This study has established a general framework for assessing the severity of depression using clinical speech; thus, a deeply time-series semantics model has been constructed. The algorithm model has significant clinical application value because face-to-face interview speech related to the HAMD-17 assessment was selected as the corpus. These were highly correlated with depressive symptoms and closely related to the assessment in real clinical practice. MGMT takes into account the multiple granularity of information as much as possible at the scene-level. Effective and differentiated information results in an accurate score for a given scene; thus the ability to provide an accurate HAMD-17 total score can be developed. The multi-task setting of MGMT has considerable potential for use in research on sub-syndromes, specific dimensions and across diagnostic symptoms, as well as in individualized purpose-oriented studies.

## Conclusion

In this study, we designed and tested an algorithmic model for depression severity classification. For clinical interview text related to depressive symptoms evaluation, considering its time-series, we proposed a Deep Learning-based NLP model based on multi-granularity and multi-task joint training, making full use of each item as well as the overall information. The test results of the proposed model demonstrate the feasibility of applying Deep Learning techniques to depressive symptoms severity assessment and have shown excellent performance. Future studies using more appropriate datasets will allow us to further improve our approach to assessing depressive symptoms. A multi-dimensional model may be a potential research direction in the future.

## Data availability statement

The original contributions presented in this study are included in this article/Supplementary material, further inquiries can be directed to the corresponding author.

## Ethics statement

The studies involving human participants were reviewed and approved by the Ethical Committee of Beijing Anding Hospital. The patients/participants provided their written informed consent to participate in this study.

## Author contributions

GW and ZH conceived the presented idea. JH supervised the project and the findings of this work. NL and LF wrote the manuscript. JW and LJ developed the model and carried out the experiment. NL and JHa collected the data. LJ and LG pre-processed and annotated the dataset. All authors contributed to the article and approved the submitted version.

## Footnotes

1
https://www.xfyun.cn/
