# Supplementary material for: Using deeply time-series semantics to assess depressive symptoms based on clinical interview speech
Source: Front Psychiatry. 2023 Feb 14;14:1104190. doi: 10.3389/fpsyt.2023.1104190 (PMC9971220; doi:10.3389/fpsyt.2023.1104190)
Supplement: Supplementary file 1 [file Table_1.DOCX]

| Appendix 1a Partial example of structured text of the doctor-patient dialogue (Original Chinese version) |
| --- |
| “0”:{  “sent”: “问：自上周以来您觉得自己情绪如何啊？答：波动比较大，呃有一次比较发很大的脾气跟孩子，然后就完了特别后悔，就情绪就就很低沉，就比较严重的抑郁状态。问：那您就是说这一周是有那种低沉的。答：对。问：嗯。呃完全是因为跟孩子发脾气这事儿，还是说没有这事儿，我的情绪也是差的？答：没这个事儿不会这么差，但是就是抑郁抑郁是长期以来是抑郁嘛，都没有特别的快乐。问：一直都处在这种状态之中？答：嗯，对。问：嗯，那家周围的家人朋友能看得出来您情绪不高吗？答：能。”,  “scene”: “抑郁情绪”,  “score”: “3”,  “score referance”: “不用语言也可以从表情姿势声音或欲哭中流露出这种情绪",  “PaAnswer”: “嗯好的一波动比较大呃有一次比较发很大的脾气跟孩子然后就完了特别后悔就情绪就就很低沉就比较严重的抑郁状态对没这个事儿不会这么差但是就是抑郁抑郁是长期以来是抑郁嘛都没有特别的快乐嗯对能”  },  “1”:{  “sent”: “问：这一周您是否会觉得自责啊？答：呃很自责。问：会觉得自己做错了什么，或者以前做过的事都是自己的错吗？答：呃这不能说都是自己的错，但是至少可能觉得百分之七八十是自己的问题啊。问：就这么说啊，会说把一些事情嗯比如说不够好，或者一些问题都归咎在自己身上吗？答：是这样。问：这有。答：对。问：那会反复思考这些事情吗？答：是的。问：那会觉得说自己现在这种生病的状态是对自己错误的惩罚吗？答：那倒不完全是，因为呃有的时候吧呃可能是一种逃避的心理，甚至有时候觉得是不是会是一种那个生理性的，呃脑子，哪有问题啊导致这样。问：但是我刚才说这种惩罚的感受是没有的？答：没有的。”  “scene”: “有罪感”,  “score”: “2”,  “score referance”: “认为自己犯了罪或反复思考以往的过失或错误”,  “PaAnswer”: “呃很自责呃这不能说都是自己的错但是至少可能觉得百分之七八十是自己的问题啊是这样对是的那倒不完全是因为呃有的时候吧呃可能是一种逃避的心理甚至有时候觉得是不是会是一种那个生理性的呃脑子哪有问题啊导致这样没有的”  }, |

| Appendix 1b Partial example of structured text of the doctor-patient dialogue (English translation) |
| --- |
| “0”:{  “sent”: “Q: What’s your mood been like this past week? A: I had a big tantrum with my children, and then I was extremely regretful and depressed, and was in a severe state of depression. Q: That is, you have that kind of low mood this week. A: Yes. Q: Hmm. Was it entirely because of the tantrum with the child, or was it that you were in a bad mood even without it? A: No, I wouldn't have been in such a bad mood, but because of the depression, that is, the long-term depression, I'm not really happy. Q: Always in this state? A: Yes, yes. Q: Okay, can the family and friends around you feel that you are not in a good mood? A: Yes.”,  “scene”: “Depressed Mood”,  “score”: “3”,  “score referance”: “Communicated non-verbally, i.e., facial expression, posture, voice, tendency to weep (persistent, moderate to severe depression)”,  “PaAnswer”: “I had a big tantrum with my children, and then I was extremely regretful and depressed, and was in a severe state of depression. Yes. No, I wouldn't have been in such a bad mood, but because of the depression, that is, the long-term depression, I'm not really happy. Yes, yes. Yes.”  },  “1”:{  “sent”: “Q: Have you been putting yourself down this past week? A: Yes, a lot of self-blame. Q: Do you feel that you did something wrong or that what you did before was your fault? A: Yes, I don't think it's all my fault, but it's my fault probably at least 70 to 80 percent. Q: Do you blame yourself extremely for things you did, the things you may not do well enough? A: Yes. Q: That happens. A: Right. Q: And do you think about these things over and over again? A: Yes. Q: Do you feel that the current state of illness is a punishment for your mistakes? A: Not exactly, it may be a kind of avoidance sometimes, and sometimes I may even think that it is a kind of physiological, or something wrong, brain and so on, which causes this. Q: What I just said about the feeling of punishment, you do not have? A: No.”,  “scene”: “Feelings Of Guilt”,  “score”: “2”,  “score referance”: “Ideas of guilt or rumination over past errors or sinful deeds ( feelings of guilt, remorse, or shame)”,  “PaAnswer”: “Yes, a lot of self-blame. I don't think it's all my fault, but it's my fault probably at least 70 to 80 percent. Yes. Right. Yes. Not exactly, it may be a kind of avoidance sometimes, and sometimes I may even think that it is a kind of physiological, or something wrong, brain and so on, which causes this. No.”  }, |
